# Supplementary material for: Quantifying the predictability of evolution by analysis of coalescent rate variation
Source: Mol Biol Evol. 2026 Aug 13;43(8):msag195. doi: 10.1093/molbev/msag195 (PMC13483991; doi:10.1093/molbev/msag195)
Supplement: Supplementary file 1 [file msag195_supplementary_data.pdf]

Supplementary Material

Quantifying the predictability of evolution by analysis of coalescent rate variation

Erik Volz Xavier Didelot

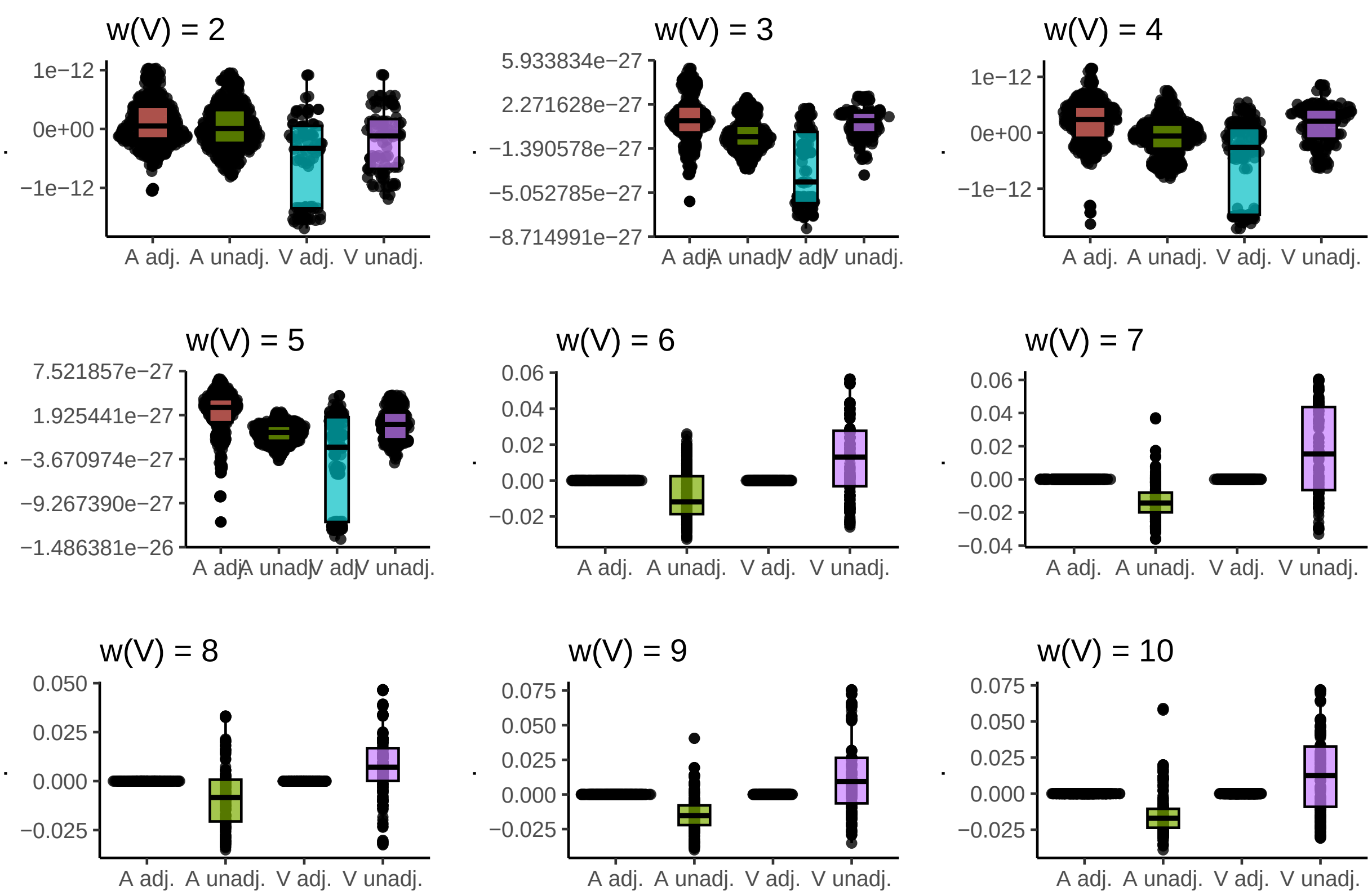

Figure S1: Comparison of coalescent odds between 1) Ancestral and variant types, and 2) with and without sample weight adjustments. Sample weights are varied from 2 to 10.

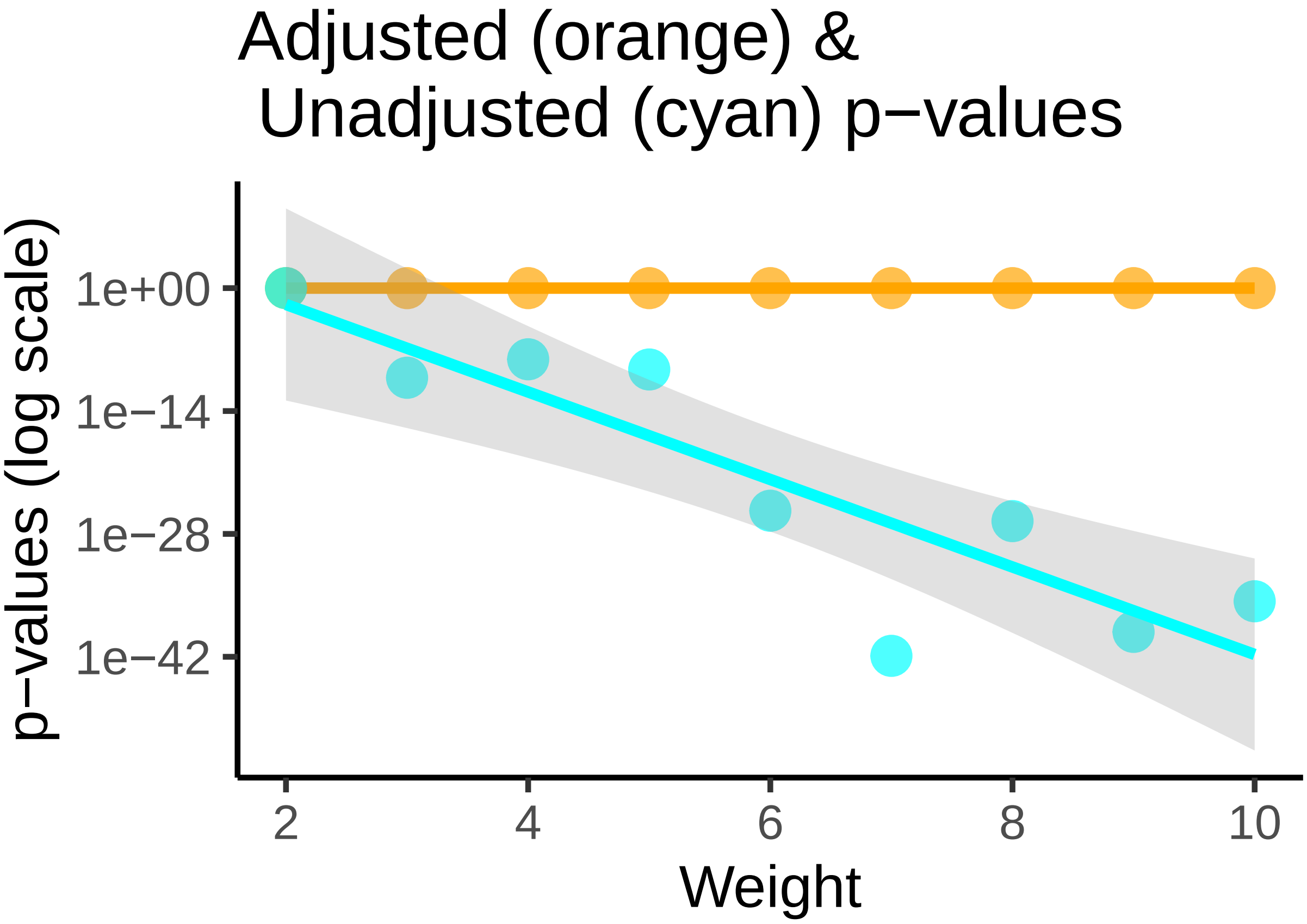

Figure S2: The p-values (log scale) of a Wilcoxon rank sum test comparing variant and ancestral coalescent odds (sampled lineages only), and comparing estimates with and without sample weighting with automatic tuning.

References

[1] Lefrancq N, Duret L, Bouchez V, Brisse S, Parkhill J, Salje H. Learning the fitness dynamics of pathogens from phylogenies. Nature. 2025;637(8046):683-90.

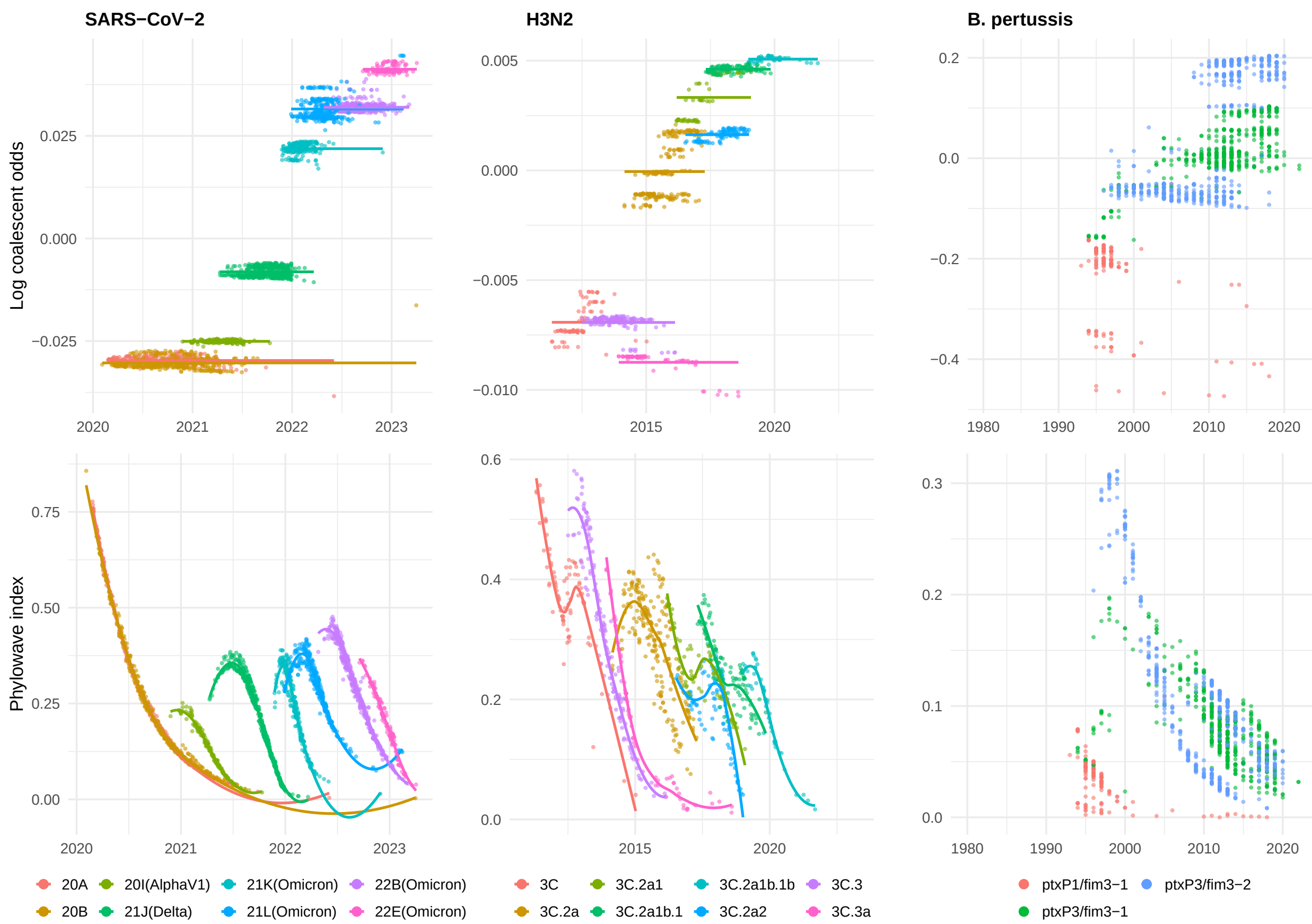

Figure S3: Comparison of coalescent odds (top, WLS approximation with default automatically tuned smoothing parameter) and the phylowave index (bottom, same parameters used in [1]). Only values from tips are shown.
